# Regulation of *CCR5* Expression in Human Placenta: Insights from a Study of Mother-to-Child Transmission of HIV in Malawi

**DOI:** 10.1371/journal.pone.0009212

**Published:** 2010-02-15

**Authors:** Bonnie R. Joubert, Nora Franceschini, Victor Mwapasa, Kari E. North, Steven R. Meshnick

**Affiliations:** 1 Department of Epidemiology, Gillings School of Global Public Health, University of North Carolina, Chapel Hill, North Carolina, United States of America; 2 College of Medicine, University of Malawi, Blantyre, Malawi; 3 Carolina Center for Genome Sciences, University of North Carolina, Chapel Hill, North Carolina, United States of America; Louisiana State University, United States of America

## Abstract

**Background:**

Human promoter polymorphisms in the chemokine co-receptor 5 gene (*CCR5*) have been noted for association with mother-to-child transmission of HIV (HIV MTCT) as well as reduced receptor expression *in vitro*, but have not been clearly associated with *CCR5* expression *in vivo*. Placental expression of *CCR5* may be influenced by such polymorphisms as well as other *in vivo* regulatory factors.

**Methodology/Principal Findings:**

We evaluated the associations between infant *CCR5* polymorphisms, measures of maternal infection, and placental expression of *CCR5* among mother-infant pairs in Blantyre, Malawi. RNA was extracted from placental tissue and used in multiplex real-time PCR to quantify gene expression. Through linear regression, we observed that *CCR5*-2554T (β = −0.67, 95% CI = −1.23, −0.11) and -2132T (β = −0.75, 95% CI = −0.131, −0.18) were significantly associated with reduced placental expression of *CCR5*. An incremental increase in *CCR5* expression was observed for incremental increases in expression of two heparan sulfate genes involved in viral infection, *HS3ST3A1* (β = 0.27, 95% CI = 0.18, 0.35) and *HS3ST3B1* (β = 0.11, 95% CI = 0.06, 0.18). Among HIV infected mothers, an incremental increase in maternal HIV viral load was also associated with higher *CCR5* expression (β = 0.76, 95% CI = 0.12, 1.39). Maternal HIV status had no overall effect (β = 0.072, 95% CI = −0.57, −0.72). Higher *CCR5* expression was observed for mothers with malaria but was not statistically significant (β = 0.37, 95% CI = −0.43, 1.18).

**Conclusions/Significance:**

These results provide *in vivo* evidence for genetic and environmental factors involved in the regulation of *CCR5* expression in the placenta. Our findings also suggest that the measurement of placental expression of *CCR5* alone is not an adequate indicator of the risk of mother-to-child transmission of HIV.

## Introduction

In sub-Saharan Africa, over 1,300,000 pregnant women were living with HIV in 2007, and more than 300,000 children are newly infected with HIV each year, primarily through mother-to-child transmission (HIV MTCT) [1]. HIV MTCT can occur during pregnancy (intrauterine transmission), during labor and delivery (intrapartum transmission), or through breastfeeding (postpartum transmission).

The chemokine (CC motif) receptor 5 (CCR5), a co-receptor of the CD4 receptor, is used by macrophage-tropic (R5) HIV-1 for cell entry [2] and is genetically regulated by the *CCR5* gene [3]. It has been demonstrated that HIV infection and progression is inhibited by competitive ligands (i.e. the chemokine RANTES) binding with the CCR5 receptor [4], [5]. Genetic variants of the *CCR5* gene such as the 32-basepair deletion in the open reading frame (*CCR5* Δ32) and promoter polymorphisms are also associated with human susceptibility to infection and/or progression of HIV-1 [2], [6], [7], [8], [9], [10], [11], [12], [13]. This is likely explained by variable receptor expression resulting from mutation [14], [15], [16].

Upregulation of *CCR5* in the placenta has been noted to increase the risk of HIV MTCT [17]. However, *CCR5* expression may be altered not only by variants in the *CCR5* gene but also by environmental factors such as maternal infection. Variability in such factors may limit the external validity of *in vivo* findings. Thus, one aim of this study was to evaluate the affects of infant *CCR5* promoter polymorphisms, maternal HIV infection, maternal HIV viral load, and maternal malaria, on *CCR5* expression in placental tissue from mother-infant pairs in Malawi.

In cells lacking the CD4 receptor, such as brain microvascular endothelial cells (BMECs), or primary genital epithelial cells (PGECs), alternative routes for HIV-1 attachment and cell entry have been suggested, including the use of heparan sulfate proteoglycans (HSPGs) [18], [19]. HSPGs are one type of proteoglycan, which is composed of a core protein (i.e. syndecan) and one or more covalently attached glycosaminoglycan (GAG) chains. HSPGs are proteoglycans with heparan sulfate (HS) attached, a highly sulfated polysaccharide made up of glucosamine and glucuronic/iduronic acid repeating disaccharide units [20]. The binding properties and thereby function of HSPGs are determined by the structure and sequence of the disaccharide units, consequential of HS biosynthesis. Various HS subtypes are produced through HS biosynthesis, which involves genetically regulated biosynthetic enzymes. One example subtype is 3-*O*-sulfated HS, synthesized by 3-*O*-sulfotransferase, which is encoded by the genes, heparan sulfate (glucosamine) 3-*O*-sulfotransferase 3A1 (*HS3ST3A1*) and B1 (*HS3ST3A1*). The 3-*O*-sulfated HS subtype has been shown to play a key role in susceptibility to herpes simplex virus -1 (HSV-1) infection *in vitro* [21], [22], [23] but has not been evaluated in the context of HIV-1 infection.

Although not specific to HS subtype, it is known that HSPGs can facilitate internalization of HIV-1 transactivator protein, Tat [24], which can induce cytokine activity and bind to heparan [25]. Treatment of cells bearing HSPGs with heparinase diminishes HIV-1 attachment and infectivity for CD4+ HeLa cells and macrophages [26], [27], an effect that was shown to differ between HIV viruses using CCR5 as a coreceptor compared to the CXCR4 coreceptor [28]. Furthermore, chemokines such as RANTES, MIP-1α, and MIP-1ß, bind not only with the CCR5 coreceptor but also with glycossaminoglycans (GAGs) bearing heparin, heparan sulfate, or chondroitin sulfate A or C [29], [30], [31]. GAGs can also strongly influence the activity of chemokines [32], [33]. Specifically, chemokines can be stored and released from T lymphocytes cytolotic granules complexed to GAGs [34], and binding with GAGs can influence chemokine structure and lead to aggregation, possibly protecting chemokines from degradation [35]. One study demonstrated that CCL5 (RANTES)-CCR5 binding-mediated apoptosis was dependent on cell-surface GAG binding and that the addition of exogenous heparin or chondroitin sulfate plus GAG digestion protected cells from apoptosis [36]. More recent studies have suggested that GAGs facilitate chemokine binding with receptors [35], [37], perhaps through electrostatic interactions [38].

It is likely that both CCR5 and HS play a role in HIV MTCT, possibly through interactions within placental tissue. Because CCR5 and HS are genetically regulated, evaluation of pertinent gene expression may provide clues for what takes place at the protein level. Thus, in addition to an evaluation of *CCR5* expression in the placenta, we quantified the expression of two key HS genes highly expressed in the placenta [39]: *HS3ST3A1* and *HS3ST3B1*, responsible for the synthesis of 3-*O*-sulfated HS. HS gene expression was evaluated for association with *CCR5* expression as well as with susceptibility to HIV MTCT in Malawi. The overall aim of this work was to describe how genetic and environmental factors may regulate *CCR5* expression in the placenta.

## Methods

### Ethics Statement

This research was approved by the University of North Carolina Institutional Review Board and the University of Malawi College of Medicine Research Ethics Committee (COMREC). Written informed consent was obtained from all study participants at the time of recruitment. Consent forms were in both English and Chichewa languages.

### Study Population

The participants were a subset of a larger cohort study of malaria and HIV in pregnancy (MHP), previously described [40], [41]. Fresh placental tissue samples from the MHP cohort were obtained from consenting study participants at delivery and immediately frozen at −80 degrees Celsius (°C). Placental tissue samples from 723 HIV positive mothers and 419 HIV negative mothers were transported to the University of North Carolina at Chapel Hill. Of the HIV positive mothers, a total of 411 samples had data on transmission status of the infant.

There were five transmission groups of interest: 1) HIV negative mother/negative infant, 2) HIV positive mother/negative infant at all visits, 3) HIV positive mother/intrauterine transmission to the infant, 4) HIV positive mother/intrapartum transmission to the infant, and 5) HIV positive mother/postpartum transmission to the infant. Power analyses indicated that a sample size of 200 would provide 80% power to detect a difference in r^2^ of 0.03 across groups. To obtain a slightly larger sample size of 250, a sample of 50 individuals was randomly selected from each of the five transmission categories, where possible. Only 47, 49, and 17 mother-infant pairs were available for intrauterine, intrapartum, or postpartum transmission events, respectively, giving a total sample size of N = 213.

### Gene Expression and Genotyping

RNA was extracted from frozen placental tissue of the 213 mother-infant pairs using RNeasy mini kit (Qiagen, Valencia, CA). In order to quantify gene expression for each RNA sample, multiplex real-time PCR was run on 96-well plates using ABI 7700 Sequence Detector (PE Biosystems) according to methods previously described [42]. A total volume of 30 µl was used, which included 10 µl of RNA and 20 µl reaction mixture [42]. The cycle conditions were 30 min at 48°C for the RT reaction, 10 min at 94°C, and then 40 temperature cycles (15 sec at 94°C and 1 min at 60°C). Relative quantification was performed where each 96-well plate was normalized to an endogenous placental RNA control sample. Negative control samples (water) were used to assess the presence of genomic DNA contamination. The difference in cycle threshold (Ct) value between a control gene, *GAPDH*, and target gene (*HS3ST3A1, HS3ST3B1*, or *CCR5*) was obtained for each sample (ΔCt). That value was subtracted from the ΔCt value of the endogenous control sample (ΔΔCt) and then transformed to a percent change in gene expression between *GAPDH* and the target gene for each sample. Infant genotyping of *CCR2*-64I and *CCR5* promoter polymorphisms were determined using a multiplex ligase detection reaction (LDR) based method with flow cytometric technology, previously described [43], [44]. Briefly, the *CCR5* promoter region containing the seven promoter SNPs and the *CCR2* open reading frame were PCR-amplified. The amplicon was probed with an upstream allele specific primer with a unique 24 nucleotide FlexMAP™TAG sequence extension (Luminex® Corporation, Austin, TX) and a downstream 5′ phosphorylated/3′ biotinylated conserved sequence primer. After allele specific hybridization, the primers were ligated, ligation products were hybridized with fluorescent bead-labeled anti-TAG probes, and the 3′ biotin group was labeled with phycoerythrin (PE). To determine genotypes, the mean fluorescence intensity of the allele-specific LDR:bead-labeled anti-TAG hybrid complexes was read on a BioPlex array reader (Bio-Rad Laboratories, Hercules, CA) into allele specific channels [43], [44].

### Statistical Analysis

The percent change in *CCR5* expression was log-transformed in order to approximate a normal distribution. Infant *CCR2*/*CCR5* SNPs were categorized into haplotypes (Table S1), based on phylogeny as previously described [45]. In order to evaluate the association between SNP/haplotype and *CCR5* expression in the placenta, linear regression was performed using log-transformed *CCR5* expression as the continuous outcome. Logistic regression was also employed to evaluate the association between SNP/haplotype and high vs. low *CCR5* expression, dichotomized at the median value. Due to small cell sizes for some polymorphisms, SNPs and haplotypes were categorized as carriers of the variant/haplotype compared to non-carriers and analyzed according to a dominant genetic model. Because the SNPs were not completely independent, exhibiting variable pairwise linkage disequilibrium (Table S2), Bonferroni correction was not employed to adjust for multiple comparisons.

The percent change in *HS3ST3A1* expression and *HS3ST3B1* expression were also log-transformed and individually evaluated for association with *CCR5* expression through linear regression, using *CCR5* expression as the continuous outcome. Measures of maternal infection including maternal HIV, maternal HIV viral load (MVL), and maternal malaria were uniquely evaluated for association with *CCR5* expression, also through linear regression.

Finally, gene expression of *CCR5, HS3ST3A1*, and *HS3ST3B1* was investigated for association with HIV MTCT through logistic regression. HIV MTCT was coded as 1 (transmission occurred) vs. 0 (no transmission). Different transmission time points (IU, IP, and PP) were evaluated in independent logistic regression models. Because gene expression did not meet the assumption of linearity of the logit, it was categorized into tertiles for this model.

Because MVL is known to be a strong predictor of HIV MTCT, it was evaluated for effect measure modification of the association between gene expression and HIV MTCT by calculating the Mantel-Haenszel test of homogeneity of the odds ratio (OR) across MVL quartiles. It was also evaluated for confounding by using the percent change in estimate criterion: |ln(CoRR)|*100>10%, where |ln(CoRR)| = |ln(crude OR–adjusted OR)|*100 [46].

## Results

### Study Population

A total of 212 mother-infant pairs evaluated for gene expression had previously been genotyped for *CCR2*/*CCR5* polymorphisms [43]. This included 154 HIV positive mothers, 103 (67%) of which had infants who became HIV positive by 12 weeks postpartum and 51 (33%) of which had HIV negative infants. Following quality control, *CCR5* SNP genotypes and placental expression was available for 196 mother-infant pairs. This included 44 (22%) HIV negative infants of HIV positive mothers, 98 (50%) HIV positive infants of HIV positive mothers, and 54 (28%) HIV negative infants of HIV negative mothers. A total of 160 mother-infant pairs also had data on HIV maternal viral load.

### CCR5 Gene Expression and CCR5 Variants

The overall mean and standard deviation (SD) of the cycle threshold values were 26.99 (SD = 4.00), 27.93 (SD = 3.92), 24.56 (2.27), and 24.51 (SD = 3.50) for *GAPDH*, *HS3ST3A1*, *HS3ST3B1*, and *CCR5*, respectively. The mean and SD of the log %change in gene expression relative to *GAPDH* expression was 5.83 (SD = 1.39), 4.63 (SD = 0.83), and 5.74 (SD = 2.02), for *HS3ST3A1*, *HS3ST3B1*, and *CCR5*, respectively. The *CCR5*-2132C→T variant was significantly associated with variable placental expression of *CCR5* (Table 1), where carriers of the T variant displayed lower placental expression of *CCR5* (mean log %change = 5.53, range = −1.61, 9.40) compared to non carriers (mean log %change = 5.87, range = 1.95, 14.55). The *CCR5*-2554 G→T variant was also significantly associated with a lower expression of *CCR5* (mean log %change for T allele = 5.69, range = −1.61, 9.91; mean log %change for G allele = 5.77, range = 1.95, 14.55), although this finding was not statistically significant in the analysis of high vs. low expression (Table 1). The minor allele frequency for *CCR5*-2132T and -2554T was 0.23 and 0.29, respectively. *CCR5* SNPs -2459G, -2135T, and -1835T corresponded to a lower risk of HIV MTCT, but these results were not statistically significant (Table 1).

**Table 1:** Frequencies and mean *CCR5* expression by *CCR5* SNP/haplotype category.

SNP/Haplotype	Genotype/# Copies£	N	Log % Change by GenotypeN, Mean (Range)	β (95% CI)†	*p*	OR (95% CI) ‡	*p*
*CCR2*-64V→I	VVVIII	1426210	131, 5,82 (−1.61, 14.55)57, 5.59 (0.69, 12.33)10, 5.36 (2.30, 7.15)	−0.27 (−0.87, 0.32)	0.374	0.69 (0.39, 1.24)	0.213
*CCR5*-2733A→G	AAAGGG	190211	177, 5.74 (−1.61, 14.55)19, 5.75 (2.56, 9.33)0, NA	0.01 (−0.95, 0.97)	0.981	0.97 (0.38, 2.51)	0.957
*CCR5*-2554G→T	GGGTTT	1029515	94, 6.09 (2.30, 14.55)89, 5.40 (−1.61, 9.91)13, 5.52 (2.77, 9.43)	−0.67 (−1.23, −0.11)	0.019	0.61 (0.35, 1.08)	0.091
*CCR5*-2459A→G	AAAGGG	5711342	53, 5.97 (−1.61, 14.55)105, 5.62 (0.69, 12.33)38, 5.76 (2.30, 9.25)	−0.32 (−0.96, 0.32)	0.324	0.62 (0.33, 1.17)	0.142
*CCR5*-2135C→T	CCCTTT	5811242	54, 6.00 (−1.61, 14.55)104, 5.62 (0.69, 12.33)38, 5.70 (2.30, 9.25)	−0.36 (−0.99, 0.28)	0.269	0.59 (0.31, 1.11)	0.104
*CCR5*-2132C→T	CCCTTT	1277114	114, 6.05 (2.30, 14.55)68, 5.09 (−1.61, 9.40)14, 6.33 (2.77, 9.43)	−0.75 (−0.131, −0.18)	0.010	0.45 (0.25, 0.81)	0.007
*CCR5*-2086A→G	AAAGGG	182283	171, 5.70 (−1.61, 14.55)25, 6.15 (3.49, 9.91)1, 3.37 (3.37, 3.37)	0.34 (−0.49, 1.18)	0.418	1.86 (0.80, 4.33)	0.149
*CCR5*-1835C→T	CCCTTT	1307112	120, 5.93 (−1.61, 14.55)65, 5.41 (0.69, 12.32)12, 5.67 (2.30, 9.25)	−0.48 (−1.05, 0.099)	0.104	0.59 (0.33, 1.05)	0.074

SNP/Haplotype	Genotype/# Copies	N	Log % Change by GenotypeN, Mean (Range)	β (95% CI)†	*p*	OR (95% CI) ‡	*p*
Haplotype A (VAGGTCAC)	012	1208111	110, 5.48 (0.69, 9.42)75, 5.82 (−1.60, 12.33)11, 7.80 (5.46, 14.55)	0.59 (0.02, 1.16)	0.042	2.08 (1.17, 3.70)	0.012
Haplotype B (VATGTCAC)	012	20570	189, 5.74 (−1.61, 14.55)7, 5.85 (3.14, 8.76)0, NA	0.12 (−1.42, 1.65)	0.880	1.47 (0.32, 6.73)	0.622
Haplotype C (VATGTCGC)	012	181283	170, 5.69 (−1.60, 14.55)25, 6.15 (3.50, 9.91)1, 3.37 (3.37, 3.37)	0.35 (−0.49, 1.19)	0.411	1.89 (0.81, 4.40)	0.141
Haplotype D (VATGTTAC)	012	1386410	125, 6.09 (2.30, 14.55)61, 5.01 (−1.61, 9.40)10, 5.88 (2.77, 9.43)	−0.96 (−1.54, −0.38)	0.001	0.36 (0.20, 0.67)	0.001
Haplotype E (VAGACCAC)	012	141656	131, 5.66 (−1.61, 14.55)59, 5.97 (1.95, 9.76)6, 5.24 (4.38, 7.48)	0.25 (−0.36, 0.85)	0.420	1.08 (0.60, 1.96)	0.802
Haplotype F1 (VAGACCAT)	012	200111	185, 5.77 (−1.61, 14.55)10, 5.17 (3.14, 9.25)1, 5.22 (5.22, 5.22)	−0.60 (−1.83, 0.64)	0.342	0.60 (0.17, 2.13)	0.432
Haplotype F2 (IAGACCAT)	012	1406210	129, 5.83 (−1.61, 14.55)57, 5.60 (0.69, 12.33)10, 5.36 (2.30, 7.15)	−0.28 (−0.88, 0.32)	0.358	0.69 (0.38, 1.24)	0.214
Haplotype G1 (VGGACCAC)	012	190211	177, 5.74 (−1.61, 14.55)19, 5.75 (2.56, 9.33)0, NA	0.01 (−0.95, 0.97)	0.981	0.97 (0.38 2.51)	0.957

† Linear regression for the association between *CCR5* expression and *CCR5* SNP/haplotype: Continuous outcome of placental expression. β: Beta coefficient, 95% CI: 95% Confidence Interval for the Beta, *p*: *p*-value.

‡ Logistic regression for the association between *CCR5* expression and *CCR5* SNP/haplotype: Dichotomous outcome of high vs. low placental expression of *CCR5* dichotomized at the median value. OR: Odds Ratio, 95% CI: 95% Confidence Interval for the Odds Ratio.

£ # Copies: Number of copies of haplotype: 0, 1, or 2 copies possible per subject. SNPs and haplotypes categorized as having one or more copies of the variant allele or haplotype compared to zero copies.

**Table 2:** Linear regression for the associations between gene expression variables.

Comparison†	β (95% CI)	*p*
*HS3ST3A1* vs. *CCR5* expression (N = 197)	0.27 (0.18, 0.35)	<0.0001
*HS3ST3B1* vs. *CCR5* expression (N = 180)	0.11 (0.06, 0.18)	<0.0001

† Continuous placental gene expression variables compared via linear regression; β: Beta coefficient; 95% CI: 95% Confidence Interval for the Beta; *p*: *p*-value.

The A haplotype, which contains the infant alleles *CCR5* -2132C and -2554G (Table S1), was associated with higher expression of *CCR5* in the placenta (Table 1). A statistically significant association in the opposite direction (lower expression of *CCR5*) was observed for haplotype D (Table 1), which contains *CCR5* -2132T and -2554T. All other alleles were the same across haplotypes A and D. Associations between *CCR5* expression and other haplotypes were not statistically significant (Table 1).

### Gene Expression Interplay in the Placenta

**Figure 1:**
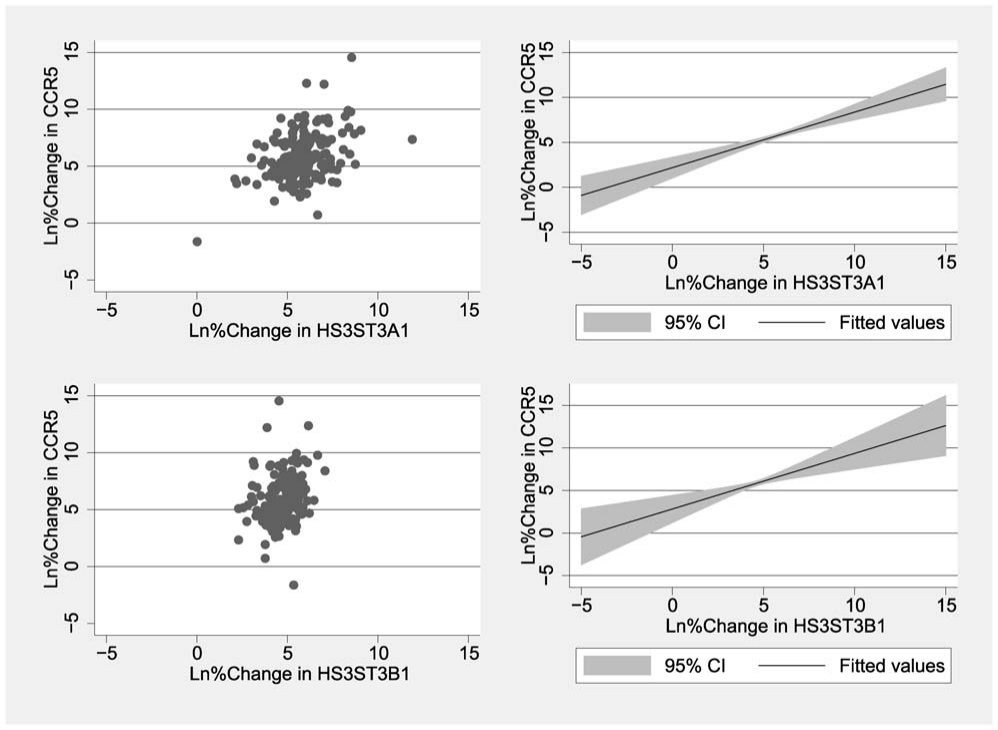
Pattern of association between placental expression of heparan sulfate genes and *CCR5*. Scatter Plots (left) and Predicted Linear Plots (right).

An interesting interplay between placental expression of *HS3ST3A1*, *HS3ST3B1*, and *CCR5* was observed in this study. An incremental increase in placental expression of *HS3ST3A1* or *HS3ST3B1* corresponded to an incremental increase in placental expression of *CCR5* (Figure 1). The positive association between placental expression of *HS3ST3A1* or *HS3ST3B1*and *CCR5* expression was statistically significant (Table 2).

**Table 3:** Association between *CCR5* placental expression and the risk of HIV MTCT at different time points.

Log %Change in Gene Expression	Intrauterine Transmission		Intrapartum Transmission		Postpartum Transmission	
	OR (95% CI)†	*p*	OR (95% CI)	*p*	OR (95% CI)	*p*
Medium low vs. Low	1.06 (0.37, 3.04)	0.907	1.11 (0.36, 3.42)	0.862	1.15 (0.25, 5.22)	0.853
Medium high vs. Low	1.02 (0.34, 3.02)	0.974	1.88 (0.60, 5.96)	0.280	0.21 (0.02, 2.18)	0.190
High vs. Low	1.25 (0.42, 3.76)	0.691	1.21 (0.36, 4.08)	0.757	1.39 (0.28, 6.83)	0.686

† Logistic regression for association between *CCR5* expression tertile and HIV MTCT (transmission vs. no transmission); OR: Odds Ratio; 95% CI: 95% Confidence Interval for the Odds Ratio; *p*: *p*-value.

### CCR5 Expression and Measures of Maternal Infection

Maternal HIV infection was not associated with *CCR5* expression in the placenta (β = 0.072, 95% CI = −0.57, −0.72, *p* = 0.826, N = 194). However, among the HIV infected mothers, maternal HIV viral load was associated with *CCR5* expression, where an incremental increase in viral load corresponded to an incremental increase in *CCR5* expression (β = 0.76, 95% CI = 0.12, 1.39; *p* = 0.020, N = 92). Maternal malaria infection also corresponded to a higher placental expression of *CCR5*, but the association was not statistically significant (β = 0.37, 95% CI = −0.43, 1.18, *p* = 0.362, N = 170).

### Gene Expression and Risk of HIV MTCT

A general trend of increasing risk of HIV MTCT with increasing expression of *CCR5* was observed but these findings were not statistically significant (Medium vs. low tertile OR = 1.16, 95% CI = 0.49, 2.73; High vs. low tertile OR = 1.26, 95% CI = 0.52, 3.03). Log maternal viral load did not modify the association between HIV MTCT and placental expression of *CCR5* (Mantel-Haenszel OR = 0.22, *p* = 0.683). In addition, MVL was not a confounder in the association based on a less than 10% change in the effect estimates after adjustment for MVL (%ln(CoRR) = 6.7%) and because MVL was not associated with *CCR5* expression (β = 0.02, 95% CI = −0.35, 0.39). Although the estimates of the association between *CCR5* expression and each transmission time point were not very precise, a similar increased risk of transmission was observed for higher expression of *CCR5* at all time points (Table 3). No significant association between the heparan sulfate genes and HIV MTCT was observed (data not shown).

### CCR5 Variants and HIV MTCT

The findings of Pedersen et al. [44] were replicated in this subset of the original cohort from Malawi, with regards to the direction of association between each *CCR5* SNP and HIV MTCT. One exception was the association between *CCR5*-1835T and the risk of HIV MTCT, which varied slightly in direction compared to previous findings (OR = 1.06 vs. RR = 0.84) [44]. Some findings also had variable statistical significance which may be a reflection of sample size (Table S3).

## Discussion

This study evaluated the regulation of *CCR5* expression in the placenta by genetic and environmental factors involved in the risk of HIV MTCT. Infant *CCR5* promoter polymorphisms -2132T and -2554T were associated with lower expression of *CCR5* in the placenta, as was the infant haplotype D, which is tagged by these alleles. Infant haplotype A was associated with significantly higher *CCR5* expression in the placenta and differs from infant haplotype D in that it contains the wild type alleles, -2132C and -2554G (Table S1) [44]. These findings provide *in vivo* evidence for *CCR5*-2554 G→T and *CCR5* -2132C→T related down regulation of *CCR5* expression in the placenta.

We expected the SNPs associated with lower *CCR5* expression to also be associated with a lower risk of HIV MTCT, and for SNPs associated with higher *CCR5* expression to be associated with a higher risk of HIV MTCT. Referring to the association between infant *CCR2*/*CCR5* SNPs and HIV MTCT previously described [44] and based on our replicate analyses, we found that this was not always the case. Notably, *CCR5* -2733G was associated with higher *CCR5* expression compared to a lower risk of HIV MTCT, although the association with expression was not statistically significant (Table S3). *CCR5* SNPs -2554T, -2132T, and -2086G also showed discrepant associations for *CCR5* expression and HIV MTCT, with variable statistical significance. The only discrepant finding that was statistically significant for both associations was observed for the D haplotype, which was associated with lower *CCR5* expression but a higher risk of HIV MTCT (Table S3). Haplotype D varies from all other haplotypes with regards to the -2132T allele, which displayed similar results (Table S3). It is possible that these discrepancies reflect smaller sample sizes or the fact that we are comparing infant genotypes/haplotypes with a combined measurement of infant and maternal placental gene expression. More expensive techniques were required to separate mother and infant tissue and we were unable to pursue this in our study. Despite this limitation, the SNP/haplotype associations suggest that predictors of *CCR5* expression do not directly correlate to the prediction of HIV MTCT and that these outcomes should be considered independently.

The discrepant SNP/haplotype associations with *CCR5* expression and HIV MTCT were partnered with the finding that *CCR5* placental expression was not associated with HIV MTCT. To obtain the best power, this association was first evaluated by using the cumulative transmission status of the infant (occurring at birth, 6 weeks, or 12 weeks postpartum), and showed no significant association. Because placenta samples were obtained at delivery, the measured *CCR5* expression was viewed to be most representative of the expression occurring during labor and delivery and thus, most relevant to the risk of IP transmission. For both cumulative and IP transmission, although the direction of effect was consistent with previous findings [17], where an increase in *CCR5* expression contributed to an increase in the risk of HIV MTCT, the association was not statistically significant. It is likely that other factors played a stronger role in predicting HIV MTCT in this study population.

Disease severity appeared to be an important regulator of placental expression of *CCR5*. Among HIV infected mothers, higher maternal HIV viral load was significantly associated with higher *CCR5* placental expression. Thus, the presence of HIV infection alone may not make as great of an impact on *CCR5* expression as does the severity of HIV disease or viral burden experienced by the individual.

Unlike maternal HIV viral load, maternal malaria was not a significant predictor of *CCR5* expression. We limited this analysis to only HIV positive mothers and found that maternal malaria did increase *CCR5* expression in the placenta but that it was not statistically significant (data not shown). Thus, we could not make any broad conclusions from the analyses with malaria. Furthermore, MVL and maternal malaria did not confound or act as effect measure modifiers in the associations between *CCR5* expression and HIV MTCT, suggesting that accounting for a key co-infection or severity of HIV infection did not explain the lack of a significant association between *CCR5* expression and HIV MTCT.

One of the most important findings from this study was the revelation of a possible interaction between *CCR5* and heparan sulfate at the genetic level. Up-regulation of *CCR5* expression in the placenta was observed at higher expression levels of two genes involved in the biosynthesis of 3-*O*-sulfated heparan sulfate, *HS3ST3A1* and *HS3ST3B1*. These findings were statistically significant and to our knowledge, are novel *in vivo* findings. It is possible that each heparan sulfate gene interacts with *CCR5* in the placenta, causing up-regulation, or that the genes share transcription regulatory regions or factors. As previously noted, heparan sulfate has been shown to interact with chemokines which bind to *CCR5*, such as RANTES, but has not been evaluated for any interaction with *CCR5* or related factors at the genetic level. Our findings press the importance of additional research on heparan sulfate and *CCR5*–related factors that may individually or cooperatively contribute to viral infection in human populations.

Overall, this study demonstrated the complexity of predicting HIV MTCT in human populations and offers new insights into regulatory factors of *CCR5* expression in the placenta. Additional epidemiological investigations are warranted in order to more clearly elucidate how *CCR5* and heparan sulfate genes may interact *in vivo* and whether combined genetic and environmental factors contribute to the risk of HIV MTCT in other populations.

## Supporting Information

Table S1 *CCR2*/*CCR5* haplotypes.
(0.04 MB DOC)

Table S2 Pairwise linkage disequilibrium for *CCR2*-64I and *CCR5* promoter polymorphisms.
(0.04 MB DOC)

Table S3 *CCR5* SNP associations with *CCR5* placental expression compared to *CCR5* SNP associations with HIV MTCT.
(0.06 MB DOC)
