# Supplementary material for: Regulation of CCR5 Expression in Human Placenta: Insights from a Study of Mother-to-Child Transmission of HIV in Malawi
Source: PLoS One. 2010 Feb 15;5(2):e9212. doi: 10.1371/journal.pone.0009212 (PMC2821402; doi:10.1371/journal.pone.0009212)
Supplement: Table S1 — CCR2/CCR5 haplotypes. (0.04 MB DOC) [file pone.0009212.s001.doc]

Table S1. *CCR2/CCR5* haplotypes

| Haplotype | *CCR2*  -64I | *CCR5*  -2733 | *CCR5*  -2554 | *CCR5*  -2459 | *CCR5*  -2135 | *CCR5*  -2132 | *CCR5*  -2086 | *CCR5*  -1835 | *CCR5*  -ORF |
| --- | --- | --- | --- | --- | --- | --- | --- | --- | --- |
| A | V | A | G | G | T | C | A | C | WT |
| B | V | A | T | G | T | C | A | C | WT |
| C | V | A | T | G | T | C | G | C | WT |
| D | V | A | T | G | T | T | A | C | WT |
| E | V | A | G | A | C | C | A | C | WT |
| F1 | V | A | G | A | C | C | A | T | WT |
| F2 | I | A | G | A | C | C | A | T | WT |
| G1 | V | G | G | A | C | C | A | C | WT |
| G2 | V | G | G | A | C | C | A | C | Δ32 |
