# Supplementary material for: Regulation of CCR5 Expression in Human Placenta: Insights from a Study of Mother-to-Child Transmission of HIV in Malawi
Source: PLoS One. 2010 Feb 15;5(2):e9212. doi: 10.1371/journal.pone.0009212 (PMC2821402; doi:10.1371/journal.pone.0009212)
Supplement: Table S2 — Pairwise linkage disequilibrium for CCR2-64I and CCR5 promoter polymorphisms. (0.04 MB DOC) [file pone.0009212.s002.doc]

Table S2. Pairwise linkage disequilibrium for *CCR2*-64I and *CCR5* promoter polymorphisms

| SNP | *CCR2*  -64I | *CCR5*  -2733 | *CCR5*  -2554 | *CCR5*  -2459 | *CCR5*  -2135 | *CCR5*  -2132 | *CCR5*  -2086 | *CCR5*  -1835 |
| --- | --- | --- | --- | --- | --- | --- | --- | --- |
| *CCR2*-64I | 1 |  |  |  |  |  |  |  |
| *CCR5*-2733 | 0.01 | 1 |  |  |  |  |  |  |
| *CCR5*-2554 | 0.10 | 0.03 | 1 |  |  |  |  |  |
| *CCR5*-2459 | 0.27 | 0.08 | 0.36 | 1 |  |  |  |  |
| *CCR5*-2135 | 0.27 | 0.08 | 0.36 | 0.98 | 1 |  |  |  |
| *CCR5*-2132 | 0.05 | 0.02 | 0.50 | 0.18 | 0.18 | 1 |  |  |
| *CCR5*-2086 | 0.03 | 0.01 | 0.26 | 0.09 | 0.09 | 0.03 | 1 |  |
| *CCR5*-1835 | 0.75 | 0.02 | 0.13 | 0.37 | 0.37 | 0.07 | 0.03 | 1 |

† Linkage disequilibrium calculated using “pwld” in *STATA* version 11.0
