# Supplementary material for: Regulation of CCR5 Expression in Human Placenta: Insights from a Study of Mother-to-Child Transmission of HIV in Malawi
Source: PLoS One. 2010 Feb 15;5(2):e9212. doi: 10.1371/journal.pone.0009212 (PMC2821402; doi:10.1371/journal.pone.0009212)
Supplement: Table S3 — CCR5 SNP associations with CCR5 placental expression compared to CCR5 SNP associations with HIV MTCT. (0.06 MB DOC) [file pone.0009212.s003.doc]

Table S3. *CCR5* SNP associations with *CCR5* placental expression compared to *CCR5* SNP associations with HIV MTCT

| SNP/Haplotype | Genotype  /#Copies£ | Association with *CCR5* expression | | Association with HIV MTCT | | | Expression vs.  HIV MTCT:  Consistent  Direction of  Association? |
| --- | --- | --- | --- | --- | --- | --- | --- |
|  |  | β (95% CI) † | Direction of  association | OR (95% CI) ‡ | Direction of  association | *PlosONE* RR  (Consistent  Direction of  Association?)¥ |  |
| *CCR2*-64V🡪I | VV  VI  II | -0.27 (-0.87, 0.32) | Lower  expression | 0.91 (0.45, 1.87) | Near the null | 1.00 (Yes) | No |
| *CCR5*-2733A🡪G | AA  AG  GG | 0.01 (-0.95, 0.97) | Near  the null | 0.24 (0.086, 0.64) | Lower risk | 0.70 (Yes) | No |
| *CCR5*-2554G🡪T | GG  GT  TT | -0.67 (-1.23, -0.11) | Lower  expression | 1.59 (0.80, 3.14) | Higher risk | 1.18 (Yes) | No |
| *CCR5*-2459A🡪G | AA  AG  GG | -0.32 (-0.96, 0.32) | Lower  expression | 0.50 (0.21, 1.14) | Lower risk | 0.78 (Yes) | Yes |
| *CCR5*-2135C🡪T | CC  CT  TT | -0.36 (-0.99, 0.28) | Lower  expression | 0.56 (0.25, 1.27) | Lower risk | 0.78 (Yes) | Yes |
| *CCR5*-2132C🡪T | CC  CT  TT | -0.75 (-0.131, -0.18) | Lower  expression | 2.58 (1.23, 5.42) | Higher risk | 1.27 (Yes) | No |
| *CCR5*-2086A🡪G | AA  AG  GG | 0.34 (-0.49, 1.18) | Higher  expression | 0.34 (0.13, 0.93) | Lower risk | 0.73 (Yes) | Yes |
| *CCR5*-1835C🡪T | CC  CT  TT | -0.48 (-1.05, 0.099) | Lower  expression | 1.06 (0.52, 2.16) | Near the null | 0.84 (No) | No |

Table S3, continued

| SNP/Haplotype | Genotype  /# Copies£ | Association with *CCR5* expression | | Association with HIV MTCT | | | Expression vs.  HIV MTCT:  Consistent  Direction of  Association? |
| --- | --- | --- | --- | --- | --- | --- | --- |
|  |  | β (95% CI) † | Direction of  association | OR (95% CI) ‡ | Direction of  association | *PLoS ONE* RR  (Consistent  Direction of  Association?)¥ |  |
| Haplotype A (VAGGTCAC) | 0  1  2 | 0.59 (0.02, 1.16) | Higher  expression | 1.89 (0.94, 3.78) | Higher risk | 1.12 (Yes) | Yes |
| Haplotype B (VATGTCAC) | 0  1  2 | 0.12 (-1.42, 1.65) | Higher  expression | 1.50 (0.15, 14.80) | Higher risk | 1.96 (Yes) | Yes |
| Haplotype D (VATGTTAC) | 0  1  2 | -0.96 (-1.54, -0.38) | Lower  expression | 2.63 (1.21, 5.72) | Higher risk | 1.24 (Yes) | No |
| Haplotype E (VAGACCAC) | 0  1  2 | 0.25 (-0.36, 0.85) | Higher  expression | 0.79 (0.39, 1.60) | Lower risk | 0.97 (Yes) | No |
| Haplotype F1 (VAGACCAT) | 0  1  2 | -0.60 (-1.83, 0.64) | Lower  expression | NA | NA | 0.55 (NA) | Yes |
| Haplotype F2 (IAGACCAT) | 0  1  2 | -0.28 (-0.88, 0.32) | Lower  expression | 0.90 (0.44, 1.85) | Near the null | 0.95 (Yes) | No |
| Haplotype G1 (VGGACCAC) | 0  1  2 | 0.01 (-0.95, 0.97) | Near  the null | 0.24 (0.09, 0.64) | Lower risk | 0.71 (Yes) | No |

† Linear regression for the association between *CCR5* expression and *CCR5* SNP/haplotype: Continuous outcome of placental expression. β: Beta coefficient, 95% CI: 95% Confidence Interval for the Beta.

‡ Logistic regression for association between *CCR5* SNPs/haplotypes and dichotomous outcome of yes/no HIV MTCT; OR: Odds Ratio; 95% CI: 95% Confidence Interval for the Odds Ratio.

£ # Copies: Number of copies of haplotype: 0, 1, or 2 copies possible per subject. SNPs and haplotypes categorized as having one or more copies of the variant allele or haplotype compared to zero copies.

¥ Replication of findings from Pedersen et al. ^46^ with regards to the direction of association. RR: Relative Risk.
